# Hospital Meetings

**Published:** 1905-12-02

**Authors:** 


### HOSPITAL MEETINGS.

### ROYAL HOSPITAL FOR INCURABLES, PUTNEY HEATH.

The annual meeting of the governors of this hospital was held on Friday, November 24, at Cannon Street Hotel, Mr. H. J. Allcroft (the treasurer) being in the chair.

The Chairman, in moving the adoption of the annual report and balance sheet, said that the financial year had been a fairly satisfactory one and the annual dinner had been a great success. The most important point to which he wished to draw their attention was the question of drainage. The drains of the hospital had been condemned, and the Board would be involved in an outlay of from £2,000 to £3,000. There was no special fund upon which they could draw for this amount and they would therefore be compelled to appeal to the public. It was absolutely necessary to have a good system of drainage, above all when the hospital contained so large a number of bedridden patients. There was also the question of the chaplain's endowment fund. Sir Massey Lopes had given another £1,000 towards this fund, which gave an assured income of £120 per annum. The sum of £220 per annum was what they required to meet the chaplain's salary and they had decided to make an appeal for the amount of £3,500 which was still needed. The chairman then announced that Mrs. Fraser McEwen, having seen that the hospital owed their bankers £3,000, had offered to give £1,000 if two other persons at that meeting would each give £1,000.

The adoption of the report having been seconded and passed, the outgoing members of the Board of Management were re-elected. A vote of thanks to the chairman and Board was moved by Mr. Daniel Stock, who inferred from the smallness of the attendance that the public was well satisfied with the manner in which the hospital was carried on. A vote of thanks to the honorary consulting physician, Dr. Goodhart, was also passed, and the proceedings then terminated.

